# Could one splicing-site single nucleotide polymorphism cause the MODY2 disease?

**DOI:** 10.1186/1758-5996-7-S1-A221

**Published:** 2015-11-11

**Authors:** Simone Brüggemann Mota, Francisco Geraldo Mello da Rocha Carvalho Neto, Danielle Rachel dos Santos Carvalho, Deborah Laredo Jezini, Tetsuo Yamane, Adolfo José da Mota

**Affiliations:** 1 Centro de Biotecnologia da Amazônia, Manaus, Brazil

## Background

Maturity-onset Diabetes of the Young (MODY; OMIM# 606391) is often related to a single gene mutation and characterized by dominant inheritance and non-insulin-dependent DM with a young age at diagnosis (Fajans et al., 2001). The most frequent subtype, MODY2, Results from SNPs in the glucokinase gene (Osback et al., 2009). Mapped to human chromosome 7p15.3-p15.1, the GCK gene consists of 10 exons and roughly 45 Kb. More than 600 SNPs were reported to this gene and among those, around 200 are related to the disease (Osback et al., 2009).

## Objective

We propose screening the GCK gene by DNA sequencing from one 83-year-old volunteer with a MODY2 diagnostic hypothesis.

## Materials and methods

Informed consent (IC) was obtained and the study was approved by the ethics committee from our institution (CAAE: 40094114.0.0000.5016/License 923.744). The total DNA was extracted using a standard protocol to the blood extraction (PuriLink® Genomic DNA Kit-InvitrogenTM by Thermo). The primers used in this study were adapted from Mota and colleagues (2011). The PCR was performed by Go®Taq Flexi DNA Polymerase kit (Promega) following the manufacturer's instructions. The amplicons sequencing was carried out using the Big Dye® Terminator v3.1 Cycle Sequencing (Applied BiosystemsTM by Life Technologies) and determined in an ABI 3500XL instrument (Applied BiosystemsTM). The sequences were analyzed using Lasergene® SeqMan ProTM for MacOs, version 11.2.1 (DNASTAR®) and were compared with the GenBank human genomic plus transcript database using the BLAST tool (Zhang et al., 2000).

## Results

**Figure 1:**
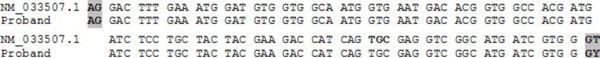
Exon 6 sequence is represented in codon-type division. Gray boxes are consensus sequence of the splicing site. NM_033507.1 is the access number to the sequence. Proband: this study. Y means C or T on that position.

In this study we were able, so far, to access information from 6 exons (including the three isoforms of exon 1, and from exon 2 to 6). The coding sequences of all exons were identical to those in the INSDC database. However, one SNP (T>C) was identified immediately after the exon 6, i. e., in the exon/intron junction, changing the consensus GT to GC (Fig. 1). To date such mutation, as presented in this study, were not reported to the MODY disease, however, it is thought that mutation within splicing site may be implicated in roughly 10% of the total disease caused by mutations (Ward and Cooper, 2010).

## Conclusion

In this study we reported one new mutation within the splicing site, downstream to the exon 6 of the GCK gene. In the future perspectives we will investigate whether this mutation is or not related to the MODY2 disease.

